# The Use of High-Throughput Transcriptomics to Identify Pathways with Therapeutic Significance in Podocytes

**DOI:** 10.3390/ijms21010274

**Published:** 2019-12-31

**Authors:** Ashish K. Solanki, Pankaj Srivastava, Bushra Rahman, Joshua H. Lipschutz, Deepak Nihalani, Ehtesham Arif

**Affiliations:** Department of Medicine, Nephrology Division, Medical University of South Carolina, Charleston, SC 29425, USA; solankia@musc.edu (A.K.S.); srivasta@musc.edu (P.S.); rahmanb@musc.edu (B.R.); lipschut@musc.edu (J.H.L.); nihalani@musc.edu (D.N.)

**Keywords:** RNA sequencing, podocytes, P53 signaling

## Abstract

Podocytes have a unique structure that supports glomerular filtration function, and many glomerular diseases result in loss of this structure, leading to podocyte dysfunction and ESRD (end stage renal disease). These structural and functional changes involve a complex set of molecular and cellular mechanisms that remain poorly understood. To understand the molecular signature of podocyte injury, we performed transcriptome analysis of cultured human podocytes injured either with PAN (puromycin aminonucleoside) or doxorubicin/adriamycin (ADR). The pathway analysis through DE (differential expression) and gene-enrichment analysis of the injured podocytes showed Tumor protein p53 (P53) as one of the major signaling pathways that was significantly upregulated upon podocyte injury. Accordingly, P53 expression was also up-regulated in the glomeruli of nephrotoxic serum (NTS) and ADR-injured mice. To further confirm these observations, cultured podocytes were treated with the P53 inhibitor pifithrin-α, which showed significant protection from ADR-induced actin cytoskeleton damage. In conclusion, signaling pathways that are involved in podocyte pathogenesis and can be therapeutically targeted were identified by high-throughput transcriptomic analysis of injured podocytes.

## 1. Introduction

Podocytes are an important cellular component of the three-layered system containing a fenestrated endothelium and glomerular basement membrane that together constitute the glomerular filtration system [1,2,3]. Podocytes have a unique architecture, consisting of a cell body that attaches to the glomerular basement membrane, and primary, secondary, and tertiary processes that wrap around the capillary in an interdigitating fashion [2]. These interdigitating podocytes are connected to the neighboring podocytes through thin membranous structures commonly known as filtration slits or slit-diaphragms, which are critical determinants of podocyte structure and function [1,2].

Many glomerular diseases, including nephrotic syndromes, diabetic nephropathy, and focal segmental glomerulosclerosis (FSGS), affect podocyte structure and function, leading to proteinuria and total loss of renal function [2,4]. Studies performed in various in vitro and in vivo disease models suggest that podocytes respond dynamically to injury through changes in their actin cytoskeleton, leading to a process known as podocyte effacement, which is characterized by the flattening of podocytes and loss of slit-diaphragm [3,5]. Although these pathological events cannot be recapitulated under in vitro conditions, the molecular processes associated with these changes have been studied using cultured human podocytes, which have served as an excellent tool to investigate the molecular and cellular mechanisms of podocyte pathogenesis [3]. Despite significant advancements in podocyte biology, the molecular mechanisms involved in podocyte pathogenesis remain poorly understood, and are the major stumbling block in identifying novel therapeutics to preserve and prevent podocyte loss during glomerular injury.

The systems biology approaches including mRNA-profiling provide a broad platform for better understanding of the complex molecular mechanisms of a disease. The information in the form of transcriptome and dynamic range RNA expression has been extensively used to investigate various diseases including podocytopathies [6,7,8]. Such analyses not only identify the molecular signature of a disease but also highlight novel signaling pathways of therapeutic significance [6,8]. To identify major pathogenic pathways in podocytes, we performed RNA-sequencing of injured podocytes and performed gene enrichment analysis. To establish the proof of principle, a major pathway enriched from this analysis was molecularly targeted and demonstrated protection of podocytes from injury.

## 2. Results

### 2.1. Transcriptional Profiling of Doxorubicin/Adriamycin (ADR)-Injured Podocytes and Identification of Signaling Pathways

**Figure 1:**
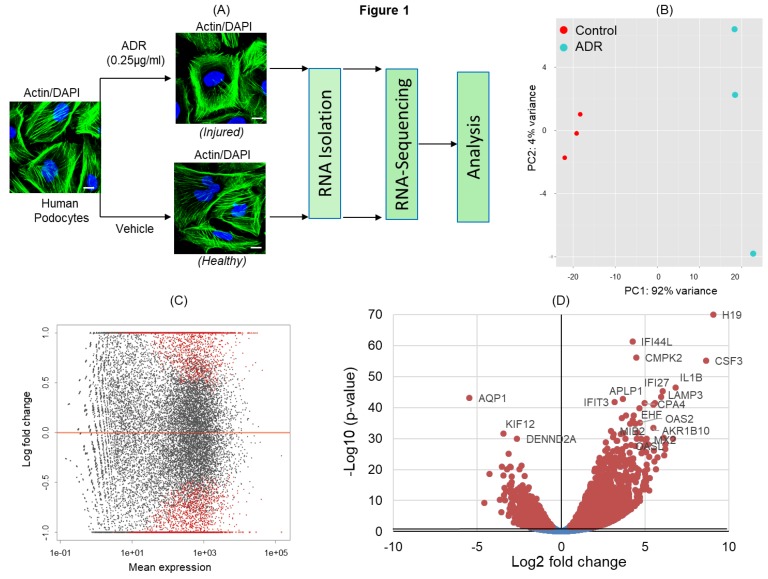
(**A**) Schematic of the experimental design for RNA-sequencing. Differentiated human podocytes treated with doxorubicin/adriamycin (ADR) or vehicle for 48 h were processed for RNA-sequencing, followed by bioinformatics analysis. 4′,6-diamidino-2-phenylindole (DAPI), scale bar = 20 µm. (**B**) Principal component analysis (PCA) plot for the RNA-sequencing gene expression data from biological replicates of control (vehicle treated) and ADR-treated human podocytes (*n* = 3). (**C**) MA plot of log2 fold changes in the gene expression versus means of normalized counts showed differentially expressed genes (DEGs) from control and ADR-treated human podocytes. DEGs with adjusted *p*-value ≤ 0.05 are represented by red dots. (**D**) The volcano plot shows all DEGs from control and ADR-treated human podocytes. The genes with maximal changes in expression are labeled.

ADR has been shown to induce severe podocyte injury under both in-vitro and in-vivo conditions [9,10]. To identify signaling pathways that are activated/deactivated in response to ADR-induced injury, cultured podocytes were treated with ADR and subjected to RNA-sequencing (Figure 1A). The principal component analysis (PCA) of transcripts across RNA datasets from control and ADR-treated cultured podocytes showed that most variability was noted between ADR-treated and healthy control groups (Figure 1B). Highest level of similarity was observed within each group in the first two components without any outliers. The DEGs (differentially expressed genes) with an adjusted *p*-values for multiple testing using Benjamini–Hochberg method (Padj ≤ 0.05) between ADR-treated and control podocytes (red dots) are shown in the MA plot (Figure 1C). Similarly, the volcano plot displays DEGs from control and ADR-treated human podocytes, where the genes with maximal changes in expression are marked (Figure 1D).

### 2.2. ADR-Induced DE (Differential Expression) Genes

**Figure 2:**
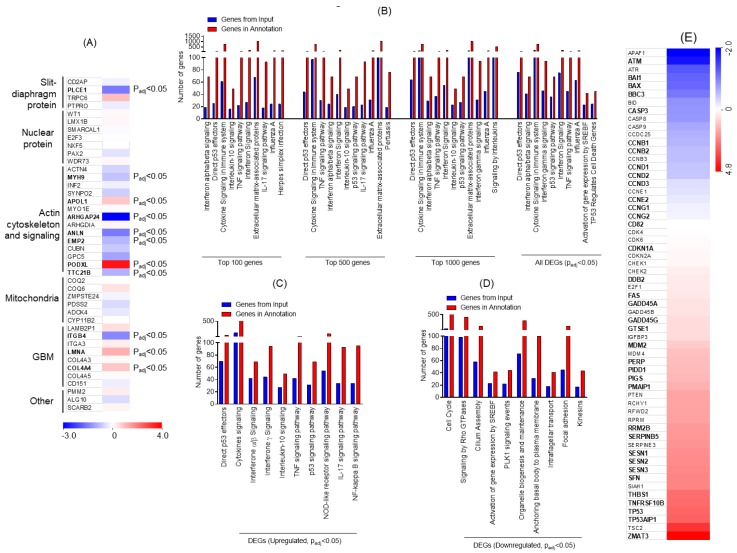
(**A**) Heat map for genes that are known to associate with nephrotic syndrome and were significantly altered upon ADR injury in podocytes, plotted from the RNA-seq data. Letters in bold denote statistical significance (Padj ≤ 0.05). (**B**) Gene Ontology (GO) analysis for the enrichment of major biological pathways was performed, and the plot was generated on the basis of statistical significance. The top 10 enriched pathways (Padj ≤ 0.05) were plotted using the DEGs. (**C**) Representation of the gene enrichment analysis of major biological pathways using the upregulated DEGs (Padj ≤ 0.05). (**D**) Representation of the gene enrichment analysis of major biological pathways using down-regulated DEGs (Padj ≤ 0.05). (**E**) Heatmap of the RNA-seq data collected from ADR-injured podocytes was plotted for genes that participate in P53 signaling and its regulation. Letters in bold denote statistical significance (Padj ≤ 0.05). GBM: glomerular basement membranes, TNF: tumor necrosis factor, IL: interleukin, p53: Tumor protein p53, NOD: nodulation factors.

The genes involved in nephrotic syndrome and associated with podocyte dysfunction were analyzed, and a heat-map was generated. The genes associated with podocyte slit-diaphragm, actin cytoskeleton, and mitochondrial and glomerular basement membranes (GBM) were DE upon ADR injury (Figure 2A). To further identify specific signaling pathways, gene enrichment analysis was performed using Topp-Gene software. Multiple pathways were enriched during ADR injury and on the basis of the statistical significance analysis, P53 was identified as the most affected signaling pathway (Figure 2B). Further stratification of data into upregulated DEGs followed by enrichment analysis showed enrichment of P53 effectors; in addition, upregulation of interferon and tumor necrosis factor (TNF) signaling pathways was noted (Figure 2C). Data from the down-regulated DEG analysis showed significant down-regulation of cell cycle; Rho-GTPase signaling; signaling by sterol regulatory element-binding protein gene (SREBF), Polo-like kinase 1 (PLK1), and organelle biosynthesis; and genes associated with focal adhesion (Figure 2D). Next, the heat-map of genes associated with P53 signaling was constructed, which showed the number of DE genes that may be involved in disease pathogenesis (Figure 2E). Collectively, these findings identified various transcriptional targets that are DE and may participate in determining podocyte health.

### 2.3. Comparative Analysis of ADR and Puromycin Aminonucleoside (PAN)-Induced Podocyte Injuries

**Figure 3:**
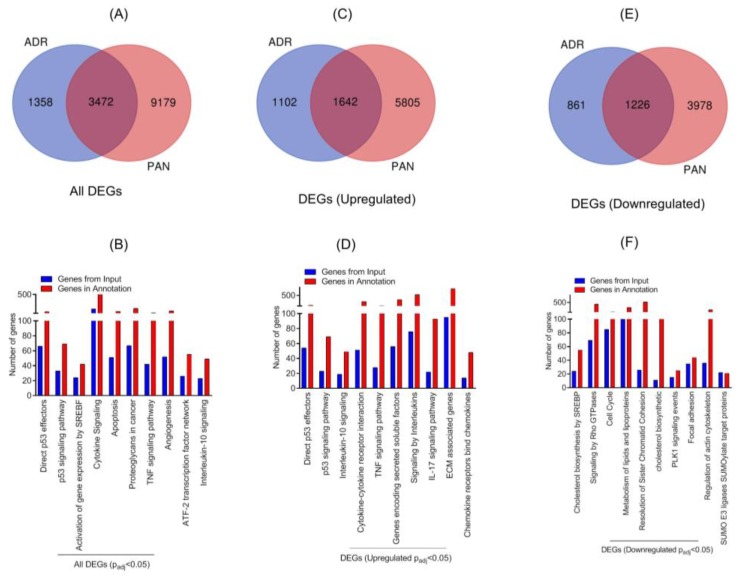
(**A**) Venn diagram of DEGs from ADR- and puromycin aminonucleoside (PAN)-injured human podocytes. (**B**) Gene enrichment analysis of the major biological pathways in both injury models was plotted (Padj ≤ 0.05). (**C**) Venn diagram showed that 1642 DEGs were common among ADR- and PAN-injured podocytes. (**D**) Gene enrichment analysis of the major biological pathways that were significantly upregulated in both injury models is presented (Padj ≤ 0.05). (**E**) Venn diagram showed 1226 common genes that were DE (differential expression) in ADR- and PAN-injured podocytes. (**F**) Gene enrichment analysis of major biological pathways down-regulated in both injury models is presented (Padj ≤ 0.05).

Our previous work has shown PAN-mediated changes in the gene expression profile of podocytes [11]. To further determine if different injuries stimulate common pathways, comparative analysis of transcriptomic data from PAN and ADR injured podocytes was performed. The Venn diagram showed 3472 DE genes that were common (Padj ≤ 0.05) in both injury models (Figure 3A). The hypergeometric test showed statistically significant *p*-value for overlapping genes (*p* < 7.125 × 10^−290^). Further, gene enrichment analysis indicated enrichment of P53 effector and signaling pathway. Among other pathways, enrichment of SREBF, cytokine, apoptosis, and TNF signaling were noted (Figure 3B). Further stratification of data showed that 1642 common DEG genes were upregulated in the two injury models (Figure 3C). The enrichment analysis of these upregulated DEGs further confirmed the upregulation of direct P53 effector, P53 signaling, cytokine signaling, and interferon and TNF signaling pathways (Figure 3D). Accordingly, 1226 common DEGs were down-regulated in the two injury models, as shown in the Venn diagram (Figure 3E). Further enrichment analysis of down-regulated genes identified signaling pathways that are involved in cholesterol biosynthesis, Rho-GTPase, cell cycle, PLK1, focal adhesion, and actin cytoskeleton (Figure 3F). Most notably, the majority of these pathways are involved in podocyte pathogenesis [1].

### 2.4. P53 Was Significantly Upregulated in Nephrotic Glomeruli

**Figure 4:**
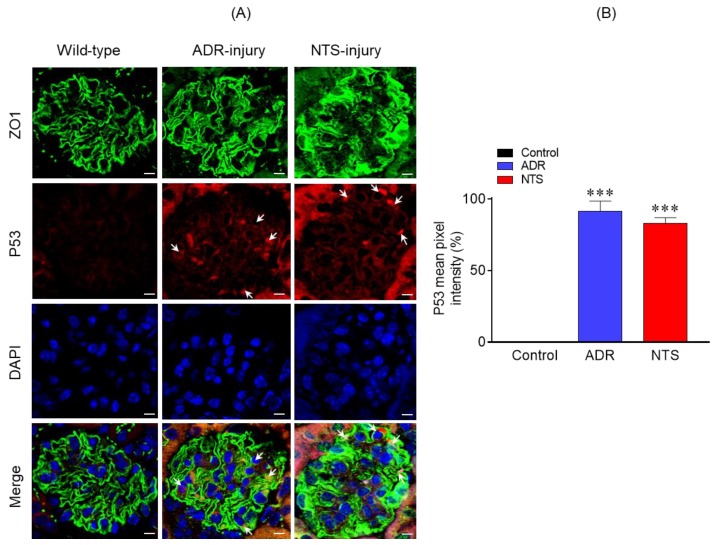
(**A**) Immunostaining analysis of kidney sections using P53 (red) and Zonula Occludens 1 (ZO1) (green) antibodies and DAPI (blue) showed increased P53 staining in the ADR- and nephrotoxic serum (NTS)-injured mice. Scale bar = 10 µm. (**B**) Quantitative analysis of mean pixel intensity showed increased P53 expression in the glomeruli of ADR- and NTS-injured mice compared to the control. A total of 30–40 glomeruli from three mice representing each group were used for statistical analyses. Data are presented as mean ± SEM; two-tailed *t*-test; *** *p* ≤ 0.001 ADR vs. control and NTS vs. control.

Because P53 signaling pathway was enriched in response to podocyte injury and has been previously associated with podocyte dysfunction [12,13], we examined the expression of P53 in the glomeruli of nephrotic mice. Therefore, P53 expression in control (uninjured) and Nephrotoxic serum (NTS)/ puromycin aminonucleoside (PAN)-injured glomeruli was analyzed using indirect immunofluorescence [12,14]. The kidney sections from these mice were immune-stained with P53 antibody and analyzed by confocal microscopy. The results showed significant increase in P53 expression in NTS- and ADR-injured glomeruli, which overlapped with the podocyte marker Zonula Occludens 1 (ZO1) (Figure 4A). In contrast, no upregulation of P53 was noted in the control glomeruli (Figure 4A). Quantitation of fluorescence intensity further confirmed increased P53 expression in the glomeruli of NTS- and ADR-injured glomeruli (Figure 4B).

### 2.5. Molecular Inhibition of P53 Signaling Prevented Damage to Podocyte Actin Cytoskeleton

**Figure 5:**
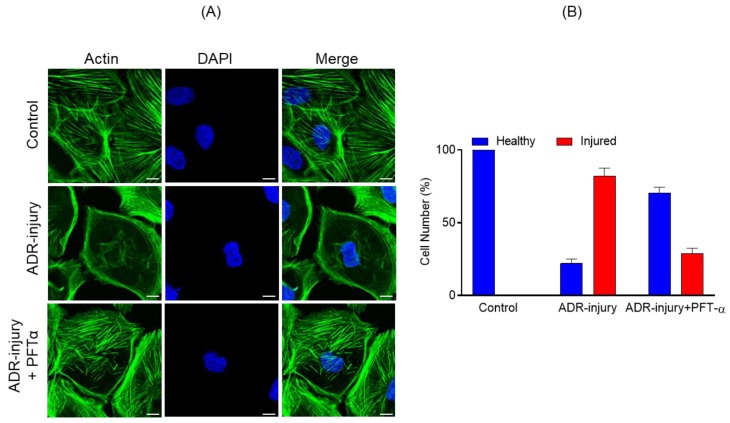
(**A**) Podocytes treated with ADR (0.25 μg/mL) for 48 h showed significant actin cytoskeleton damage (green) with accumulation of actin stress fibers at the cell periphery. In contrast, significant recovery of actin cytoskeletal organization was noted in Pifithrin-α (PFT-α)-treated podocytes. Scale bar = 20 µm. (**B**) The quantitative analysis showed >50% increase in the number of healthy podocytes with a concomitant decrease in ADR injured podocytes upon PFT-α treatment; in contrast, minimal recovery was observed in the vehicle-treated podocytes.

Because P53 signaling was most enriched during podocyte injury, we hypothesized that inhibiting P53 signaling may have a protective effect. To test this hypothesis, we used pifithrin-α, a known inhibitor of P53-dependent gene transcription including cyclin G, p21/waf1, and mdm2 expression [15]. Human podocytes incubated with ADR (0.25 μg/mL) for 48 h along with pifithrin-α (10 µM) or vehicle were analyzed by immunostaining. Although ADR treatment induced actin cytoskeleton disorganization, as reported previously [9,16], the actin cytoskeleton of ADR-injured podocytes treated with pifithrin-α was well-preserved and showed minimal disorganization (Figure 5A). Further, quantification of actin cytoskeleton changes showed >50% podocytes with healthy morphology when treated with pifithrin-α (Figure 5B).

## 3. Discussion

Several proteins and signaling pathways contribute to the unique morphology of podocytes, which is central to their function [1,2]. Understanding their contribution is critical for deciphering the pathogenic mechanisms that participate in podocyte dysfunction. In the past, high-throughput analyses have been used to understand the molecular mechanisms and pathways that are involved in podocyte pathogenesis [17,18]. Recent advancements in the RNA-sequencing approach have provided a better platform to understand the complexity of a transcriptome and dynamic ranges of RNA expression with minimal noise [6,7]. In this study, we used RNA-sequencing and bioinformatics analyses to identify novel signaling pathways that participate in podocyte pathogenesis.

Our bioinformatic analysis showed that ADR- and/or PAN-induced injury to podocytes led to the enrichment of P53 effector and signaling genes that may participate in the maintenance of podocytes cellular homeostasis [13,19]. The persistent activation of p53 that is observed in diseases such as diabetic nephropathy and nephrotic syndromes results in cellular apoptosis and leads to progressive loss of renal function [13,19]. Accordingly, the inhibition of P53 attenuated PAN-induced apoptosis [12]. To further substantiate and validate our bioinformatics data, we performed a comparative analysis of ADR- and PAN-injured podocytes, which showed enrichment of P53 effector and signaling genes, indicating the role of P53 in podocyte pathogenesis [12]. In a similar fashion, the molecular inhibition of P53 was found to prevent ADR-injured actin cytoskeleton damage (Figure 5). Further analysis of upregulated DEGs identified immunological pathways including interleukin and cytokine signaling, interferon and TNF signaling. The role of IL-6 in podocyte pathogenesis has been shown previously, where high glucose and IL-6 stimulation led to podocyte hypertrophy, which was attenuated by anti-IL-6-neutralizing antibodies [20]. Similarly, other cytokines such as IL17A, interferon (IFN)-α, and IFN-β were found to be involved in glomerulosclerosis [21,22]. Studies further indicate that podocytes possess components of innate immunity and their induction may contribute to podocyte injury and podocytopathies [23].

Further analysis of down-regulated DEGs identified cholesterol synthesis and associated pathways. Sterol regulatory element-binding proteins (SREBPs) are known to directly activate the expression of genes involved in the synthesis and uptake of cholesterol, fatty acids, triglycerides, phospholipids, and cofactor nicotinamide adenine dinucleotide phosphate (NADPH) [24]. The podocytes contain lipid raft domains that are rich in cholesterol and contribute towards the critical regulatory functions such as membrane fluidity, membrane protein trafficking, and the assembly of signaling molecules [5]. It is also known that the silt-diaphragm protein podocin binds and recruits cholesterol that is required for its function [25]. Recent findings also suggest that cholesterol homeostasis is critical for podocyte function through a regulatory mechanism of influx and efflux, whose alteration may lead to podocyte damage [25]. The other significantly down-regulated DGEs included Rho-GTPase, which plays a critical role in regulating podocyte actin dynamics and cell migration [26]. The inactivation of Rho-GTPase was shown to be associated with hyper motility, and its activation leads to a decrease in cellular motility [26,27]. Cell cycle was the third highly downregulated occurrence, whose aberrant progression has been noted in many glomerular diseases and is often accompanied by cellular hypertrophy [28]. Additionally, the pathways involved in actin cytoskeleton organization and focal adhesion were downregulated, which is known to induce podocyte dysfunction [29].

To further validate the concepts presented in this study, we tested the molecular inhibition of P53 in nephrotic mouse models, where it is significantly upregulated [12,14]. Strikingly, P53 inhibition protected podocytes from injury-induced actin cytoskeleton damage. These results provided support for our hypothesis that this approach can be utilized in identifying novel signaling pathways with high therapeutic significance. Further advancement of this approach can be achieved through simultaneous targeting of multiple pathways to induce a synergistic effect in preventing podocyte loss, thus promoting the concept of combination therapy.

## 4. Materials and Methods

### 4.1. Podocyte Cell Culture

Human podocytes were cultured as described previously [3,30,31]. In brief, these cells were cultured in Roswell Park Memorial Institute-1640 (RPMI-1640)-based medium supplemented with 10% fetal bovine serum (FBS; Invitrogen), 2g/L of NaHCO_3_, insulin-transferrin-selenium (Sigma-Aldrich, USA, St. Louis, MO, USA), and 200 units/mL penicillin and streptomycin (Roche) [3,30]. The human podocytes were grown on collagen-coated culture dishes at 33 °C and 5% CO_2_, and were differentiated by thermo switching to 37 °C and by removing insulin-transferrin-selenium from the media, as described previously [3,30,31]. Podocyte injuries were developed using adriamycin (ADR) (0.25 μg/mL) for 48 h in serum-free media, whereas control podocytes were incubated only in the serum-free media [9]. The ADR-injured and control podocytes were processed for RNA isolation. In a similar fashion, the drug-induced recovery was initiated by adding Pifithrin-α (PFT-α) or vehicle in serum-free medium along with ADR.

### 4.2. RNA-Seq

Differentiated human podocytes were treated with ADR (0.25 μg/mL) for 48 h. The control and ADR-injured podocytes were lyzed, and RNA isolation was performed using the Qiagen RNA isolation kit according to the manufacturer’s instructions. All experiments were performed in triplicate. RNA samples were submitted for RNA sequencing and bioinformatics analysis to the Bioinformatics Shared Resource Core facility of the Medical University of South Carolina (MUSC). Illumina HiScanSQ (USA, San Diego, CA, USA) was used for the RNA sequencing, as described previously [32]. Briefly, RNA quality and integrity was tested on an Agilent 2200 Tape Station (Agilent Technologies, USA, Santa Clara, CA, USA). RNAseq libraries were prepared using the TruSeq RNA Sample Prep kit as per the manufacturer’s instructions (Illumina, USA, San Diego, CA) using a total RNA of about 100–200 ng. Bioinformatics analysis was performed at the bioinformatics core facility of MUSC, as described previously [32]. Trimmomatic program was used to process the data, and the sequences were confirmed using FastQC and aligned with human genome build HG19 using Tophat (Bowtie2). DESeq2 R package was used for DE analysis. The *p*-values were adjusted using the Benjamini–Hochberg’s approach and adjusted *p*-value < 0.05 assigned as differential expressing genes (DEGs). All the raw and process data were submitted to GEO database (GEO Accession # GSE124622).

### 4.3. Bioinformatics

Functional enrichment analysis was performed using the online software ToppGene (https://toppgene.cchmc.org/enrichment.jsp), which is a one-step portal for the enrichment analysis based on functional annotations and protein interactions network [33]. Calculations were performed using probability density function, and *p*-values were corrected using false discovery rate (FDR), with a *p*-value ≤ 0.05 being the cutoff value for analysis. Venn diagrams were plotted using the online software developed by Bioinformatics and Evolutionary Genomics (http://bioinformatics.psb.ugent.be/webtools/Venn/) to identify common gene set between the two different injury models. Hypergeometric test was performed to evaluate the significance of overlapping genes using the online program (http://nemates.org/MA/progs/overlap_stats.html).

### 4.4. Mouse Models of Glomerular Injury

Mice with C57BL/6N genetic background were treated with 80 µl nephrotoxic serum (NTS) (Probetex Inc, San Antonio, TX, PTX-001) through retro-orbital injection. Proteinuria in these mice initiated at 4 h, and persisted until 7 days, after which the kidneys were harvested for immune-histological analysis [11]. Similarly, an ADR-induced chronic model of glomerular injury was generated in BALB/c genetic background mice by injecting 7 mg/kg ADR (doxorubicin hydrochloride) retro-orbitally, which showed significant proteinuria at 4 weeks [11]. Kidneys were harvested at 4 weeks, and immuno-histological analyses were performed using specific antibodies.

### 4.5. Immuno-Fluorescence Staining

Control and experimental (ADR and NTS) nephrotic mouse models were perfused with 4% paraformaldehyde prepared in phosphate-buffered saline, followed by isolating their kidneys. These kidney samples were processed for paraffin embedding, and 5 μm sections were obtained with a sliding microtome. Kidney tissue sections were deparaffinized and incubated with Tris-EDTA (pH = 9.0) buffer for antigen retrieval at 65 °C overnight and blocked with 5% BSA (in 1X-Tris Buffered Saline) for 1 h at room temperature. Primary antibodies for ZO1 (Invitrogen, catalog # 61-7300) (1:100 dilution) and P53 (Santa Cruz # p53 antibody (DO-1): sc-126) (1:100 dilution) were diluted in 3% bovine serum albumin (in 1XPBS) and incubated overnight at 4 °C. The sections were washed five times with 1XTBS and then incubated with Alexa 488 Fluor-labeled secondary antibody (catalogue number # A-11008; Thermo-Fisher) and/or Alexa Fluor 568/594 goat anti-mouse IgG(H + L) (catalogue number # A-11004/# A-11005, Thermo-Fisher) was used at a dilution of 1:1000 for 1 h at 37 °C. Kidney sections were mounted with 4′,6-diamidino-2-phenylindole (DAPI) and left overnight in the dark for drying. Sections stained with secondary antibodies alone were used as negative controls. All animal studies were approved under the protocol number # IACUC-2018-00360 by the MUSC-IACUC (Institutional Animal Care and Use Committee) and were conducted as per the National Institutes of Health (NIH) guidelines for Care and Use of Laboratory Animals. Human podocytes grown on coverslips were treated with ADR with/without PFT-α and were fixed with 4% paraformaldehyde (in 1XPBS) after 48 h of treatment, followed by permeabilization with 0.1% Sodium dodecyl sulfate (SDS). Podocytes were incubated for 1 h with Alexa-488-phalloidin at room temperature. After incubation, the coverslips were washed and mounted using Gel-Mount.

### 4.6. Indirect Immunofluorescence Microscopy

Fluorescence microscopy was performed using the Leica fluorescence (Leica-DMI4000B) microscope (Leica Microsystems Inc, Wetzlar, Germany) equipped with Leica Application Suite X acquisition software, and the images were captured using 40× oil objective. Confocal microscope was used to image the kidney sections, which was performed using an Olympus FluoView FV10i-LIV microscope (Olympus Corporation, Shinjuku, Tokyo, Japan) equipped with Olympus FluoView acquisition software ver. 4.2, and the images were captured using 60× water immersion objective. The laser excitations used were 405, 473, 559, and 635 nm. All parameters, including exposure time, were kept constant throughout the image capturing. Images were later processed and analyzed using Image-J software. The mean pixel intensity of P53 expression in the glomeruli were obtained by keeping a constant threshold and selecting the regions of interest. Statistical analyses were performed using GraphPad Prism8 software and a *p* ≤ 0.05 was considered statistically significant. Kruskal–Wallis test (nonparametric test) was used for the analysis of statistical significance.

## References

[1] Pavenstadt H., Kriz W., Kretzler M. (2003). Cell biology of the glomerular podocyte. Physiol. Rev..

[2] Tryggvason K., Patrakka J., Wartiovaara J. (2006). Hereditary proteinuria syndromes and mechanisms of proteinuria. N. Engl. J. Med..

[3] Arif E., Rathore Y.S., Kumari B., Ashish F., Wong H.N., Holzman L.B., Nihalani D. (2014). Slit diaphragm protein Neph1 and its signaling: A novel therapeutic target for protection of podocytes against glomerular injury. J. Biol. Chem..

[4] Rosenberg A.Z., Kopp J.B. (2017). Focal Segmental Glomerulosclerosis. Clin. J. Am. Soc. Nephrol. CJASN.

[5] Shankland S.J. (2006). The podocyte′s response to injury: Role in proteinuria and glomerulosclerosis. Kidney Int..

[6] Costa V., Aprile M., Esposito R., Ciccodicola A. (2013). RNA-Seq and human complex diseases: Recent accomplishments and future perspectives. Eur. J. Hum. Genet. EJHG.

[7] Casamassimi A., Federico A., Rienzo M., Esposito S., Ciccodicola A. (2017). Transcriptome Profiling in Human Diseases: New Advances and Perspectives. Int. J. Mol. Sci..

[8] Rudnicki M., Beckers A., Neuwirt H., Vandesompele J. (2015). RNA expression signatures and posttranscriptional regulation in diabetic nephropathy. Nephrol. Dial. Transplant..

[9] Xu W., Ge Y., Liu Z., Gong R. (2014). Glycogen synthase kinase 3beta dictates podocyte motility and focal adhesion turnover by modulating paxillin activity: Implications for the protective effect of low-dose lithium in podocytopathy. Am. J. Pathol..

[10] Arif E., Solanki A.K., Nihalani D. (2016). Adriamycin susceptibility among C57BL/6 substrains. Kidney Int..

[11] Arif E., Solanki A.K., Srivastava P., Rahman B., Fitzgibbon W.R., Deng P., Budisavljevic M.N., Baicu C.F., Zile M.R., Megyesi J. (2019). Mitochondrial biogenesis induced by the β2-adrenergic receptor agonist formoterol accelerates podocyte recovery from glomerular injury. Kidney Int..

[12] Wada T., Pippin J.W., Nangaku M., Shankland S.J. (2008). Dexamethasone′s prosurvival benefits in podocytes require extracellular signal-regulated kinase phosphorylation. Nephron Exp. Nephrol..

[13] Fukuda R., Suico M.A., Kai Y., Omachi K., Motomura K., Koga T., Komohara Y., Koyama K., Yokota T., Taura M. (2016). Podocyte p53 Limits the Severity of Experimental Alport Syndrome. J. Am. Soc. Nephrol. JASN.

[14] Deshpande S.D., Putta S., Wang M., Lai J.Y., Bitzer M., Nelson R.G., Lanting L.L., Kato M., Natarajan R. (2013). Transforming growth factor-beta-induced cross talk between p53 and a microRNA in the pathogenesis of diabetic nephropathy. Diabetes.

[15] Sohn D., Graupner V., Neise D., Essmann F., Schulze-Osthoff K., Janicke R.U. (2009). Pifithrin-alpha protects against DNA damage-induced apoptosis downstream of mitochondria independent of p53. Cell Death Differ..

[16] Zhou S., Wang P., Qiao Y., Ge Y., Wang Y., Quan S., Yao R., Zhuang S., Wang L.J., Du Y. (2016). Genetic and Pharmacologic Targeting of Glycogen Synthase Kinase 3beta Reinforces the Nrf2 Antioxidant Defense against Podocytopathy. J. Am. Soc. Nephrol. JASN.

[17] Jiang L., Hindmarch C.C., Rogers M., Campbell C., Waterfall C., Coghill J., Mathieson P.W., Welsh G.I. (2016). RNA sequencing analysis of human podocytes reveals glucocorticoid regulated gene networks targeting non-immune pathways. Sci. Rep..

[18] Fu J., Wei C., Lee K., Zhang W., He W., Chuang P., Liu Z., He J.C. (2016). Comparison of Glomerular and Podocyte mRNA Profiles in Streptozotocin-Induced Diabetes. J. Am. Soc. Nephrol. JASN.

[19] Wen Y., Zhou P., Liu L., Wang Z., Zhang Y., Liang J. (2016). Effect of the knockdown of Cabin1 on p53 in glomerular podocyte. J. Recept. Signal Transduct. Res..

[20] Jo H.A., Kim J.Y., Yang S.H., Han S.S., Joo K.W., Kim Y.S., Kim D.K. (2016). The role of local IL6/JAK2/STAT3 signaling in high glucose-induced podocyte hypertrophy. Kidney Res. Clin. Pract..

[21] Yan J., Li Y., Yang H., Zhang L., Yang B., Wang M., Li Q. (2018). Interleukin-17A participates in podocyte injury by inducing IL-1beta secretion through ROS-NLRP3 inflammasome-caspase-1 pathway. Scand. J. Immunol..

[22] Migliorini A., Angelotti M.L., Mulay S.R., Kulkarni O.O., Demleitner J., Dietrich A., Sagrinati C., Ballerini L., Peired A., Shankland S.J. (2013). The antiviral cytokines IFN-alpha and IFN-beta modulate parietal epithelial cells and promote podocyte loss: Implications for IFN toxicity, viral glomerulonephritis, and glomerular regeneration. Am. J. Pathol..

[23] Xia H., Bao W., Shi S. (2017). Innate Immune Activity in Glomerular Podocytes. Front. Immunol..

[24] Horton J.D., Goldstein J.L., Brown M.S. (2002). SREBPs: Activators of the complete program of cholesterol and fatty acid synthesis in the liver. J. Clin. Investig..

[25] Merscher S., Pedigo C.E., Mendez A.J. (2014). Metabolism, energetics, and lipid biology in the podocyte—cellular cholesterol-mediated glomerular injury. Front. Endocrinol..

[26] Kistler A.D., Altintas M.M., Reiser J. (2012). Podocyte GTPases regulate kidney filter dynamics. Kidney Int..

[27] Wang L., Ellis M.J., Gomez J.A., Eisner W., Fennell W., Howell D.N., Ruiz P., Fields T.A., Spurney R.F. (2012). Mechanisms of the proteinuria induced by Rho GTPases. Kidney Int..

[28] Hagen M., Pfister E., Kosel A., Shankland S., Pippin J., Amann K., Daniel C. (2016). Cell cycle re-entry sensitizes podocytes to injury induced death. Cell Cycle.

[29] Mundel P., Shankland S.J. (2002). Podocyte biology and response to injury. J. Am. Soc. Nephrol. JASN.

[30] Saleem M.A., Ni L., Witherden I., Tryggvason K., Ruotsalainen V., Mundel P., Mathieson P.W. (2002). Co-localization of nephrin, podocin, and the actin cytoskeleton: Evidence for a role in podocyte foot process formation. Am. J. Pathol..

[31] Saleem M.A., O′Hare M.J., Reiser J., Coward R.J., Inward C.D., Farren T., Xing C.Y., Ni L., Mathieson P.W., Mundel P. (2002). A conditionally immortalized human podocyte cell line demonstrating nephrin and podocin expression. J. Am. Soc. Nephrol. JASN.

[32] Mortazavi A., Williams B.A., McCue K., Schaeffer L., Wold B. (2008). Mapping and quantifying mammalian transcriptomes by RNA-Seq. Nat. Methods.

[33] Chen J., Bardes E.E., Aronow B.J., Jegga A.G. (2009). ToppGene Suite for gene list enrichment analysis and candidate gene prioritization. Nucleic Acids Res..

